# *Voacanga globosa* Spirobisindole Alkaloids Exert Antiviral Activity in HIV Latently Infected Cell Lines by Targeting the NF-κB Cascade: In Vitro and In Silico Investigations

**DOI:** 10.3390/molecules27031078

**Published:** 2022-02-05

**Authors:** Ma. Sheila M. de Jesus, Allan Patrick G. Macabeo, John Donnie A. Ramos, Von Novi O. de Leon, Kaori Asamitsu, Takashi Okamoto

**Affiliations:** 1 The Graduate School, University of Santo Tomas, España Blvd., Manila 1015, Philippines; jaramos@ust.edu.ph; 2 Department of Biological Sciences, College of Science, University of Santo Tomas, España Blvd., Manila 1015, Philippines; vonnovi.deleon.sci@ust.edu.ph; 3 Laboratory for Organic Reactivity, Discovery and Synthesis (LORDS), Research Center for Natural and Applied Sciences, University of Santo Tomas, España Blvd., Manila 1015, Philippines; agmacabeo@ust.edu.ph; 4 Molecular Diagnostics and Therapeutics Laboratory, Research Center for Natural and Applied Sciences, University of Santo Tomas, España Blvd., Manila 1015, Philippines; 5 Department of Molecular and Cellular Biology, Graduate School of Medical Sciences, Nagoya City University, Nagoya 4678601, Japan; asamitsu@med.nagoya-cu.ac.jp (K.A.); takoka221@gmail.com (T.O.)

**Keywords:** spirobisindole alkaloids, globospiramine, NF-ĸB, anti-HIV, HIV latency, *Voacanga globosa*

## Abstract

Since the efficiency in the transcription of the HIV genome contributes to the success of viral replication and infectivity, we investigated the downregulating effects of the spirobisindole alkaloids globospiramine (**1**), deoxyvobtusine (**2**), and vobtusine lactone (**3**) from the endemic Philippine medicinal plant, *Voacanga globosa*, during HIV gene transcription. Alkaloids **1**–**3** were explored for their inhibitory activity on TNF-α-induced viral replication in two latently HIV-infected cell lines, OM10.1 and J-Lat. The induction of HIV replication from OM10.1 and J-Lat cells elicited by TNF-α was blocked by globospiramine (**1**) within noncytotoxic concentrations. Furthermore, globospiramine (**1**) was found to target the NF-ĸB activation cascade in a dose-dependent manner when the transcriptional step at which inhibitory activity is exerted was examined in TNF-α-induced 293 human cells using transient reporter (luciferase) gene expression systems (HIV LTR-luc, ĸB-luc, and mutant ĸB-luc). Interrogation through molecular docking against the NF-ĸB p50/p65 heterodimer and target sites of the subunits comprising the IKK complex revealed high binding affinities of globospiramine (**1**) against the S281 pocket of the p65 subunit (BE = −9.2 kcal/mol) and the IKKα activation loop (BE = −9.1 kcal/mol). These findings suggest globospiramine (**1**) as a molecular inspiration to discover new alkaloid-based anti-HIV derivatives.

## 1. Introduction

Infection by human immunodeficiency virus (HIV) displays a spectrum of signs and symptoms that escalates to damage the immune system, leading to Acquired Immune Deficiency Syndrome (AIDS). Around 37.9 million people are living with HIV (PLHIV), 23.3 million of which received antiretroviral therapy (ART) at the end of 2020 [1]. In the Philippines, where there is an alarming increase in HIV infections, cumulative data from 1984 to August 2021 showed 53,286 PLHIV are on ART [2]. The current antiretroviral drugs target viral enzymes, including reverse transcriptase, integrase, and protease, and it is carried out usually in combination [3,4], which has been generally effective in treating HIV infections with a low likelihood of progressing to AIDS [5]. However, high morbidity and mortality rates still outpace the treatment response on a global scale because of (1) limited accessibility to antiretroviral drugs, often limited to average- to high-income countries [6], (2) drug resistance due to viral mutations [7], (3) adverse reactions [8], and (4) a latency shift in the cellular reservoirs [9]. The biological complexity of HIV can be reduced if not eliminated by having drugs target the viral gene expression that should prevent viral production and replication. This target remains elusive in drug discovery.

The latency shift exhibited by cellular reservoirs of HIV is a reversible state of infection in which inactive, integrated provirus particles are retained within the host cell while evading the host immune response [10]. The interference of HIV transcriptional mechanisms and chromatin molecular environment suppress replication and gene expression in HIV-1 latently infected resting CD4 + T cells. In active infected cells, the transcription factors, nuclear factor κB (NF-κB), nuclear factor of activated T cells (NFAT), and Sp1, are recruited into the HIV long terminal repeats to initiate viral replication, while Tat initiates transcriptional elongation, which is regulated by the positive transcription elongation factor b (P-TEFb) [9,11,12,13]. Insufficient expression and inactivity of these factors block successful replication and are related to latency shift [9,11,12]. In addition, NF-κB is widely established in HIV latently infected cells and functions in the early phase of HIV transcription, therefore emerging as a target of anti-HIV chemotherapy [14,15]. Moreover, upstream signaling proteins that eventually activate NF-κB are targeted by pharmacological compounds such as bryologs, phorbol esters, and alkaloids [15,16,17].

The renewed interest in alkaloidal natural products is undeniable, because they are considered excellent sources and inspirations for novel derivatives against HIV [18]. For example, the alkaloids corydine and norisoboldine from *Croton echinocarpus* [19] and emetine from the roots of ipecac inhibited viral reverse transcriptase activity [20]; polycitone A, an alkaloid from marine ascidians or sea squirts, inhibited polymerase activity [21]; derivatives of aloperine, a plant alkaloid, inhibited HIV-1 entry [22]; an alkaloid extracted from the aerial parts of orange bulbine inhibited HIV protease [23]; and an analog of cortistatin A, a natural marine steroidal alkaloid, blocked HIV transcription by suppressing the trans-activator of the transcription or Tat [24,25].

**Figure 1:**
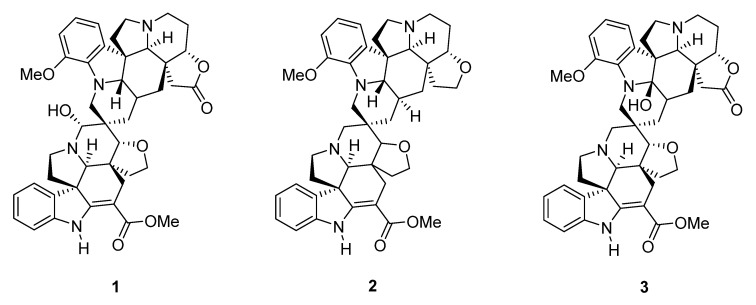
Spirobisindole alkaloids globospiramine (**1**), deoxyvobtusine (**2**), and vobtusine lactone (**3**) from *Voacanga globosa*.

Indole alkaloids are structurally diverse secondary metabolites endowed with a wide range of biological activities [26,27,28]. These alkaloids and their derivatives are known to target HIV enzymes in the viral life cycle, such as reverse transcriptase, polymerase integrase, and protease [29]. In this study, we explored the in vitro anti-HIV activity of three spirocyclic bisindole alkaloids from *Voacanga globosa* (Merr.) Blanco, namely globospiramine (**1**), deoxyvobtusine (**2**), and vobtusine lactone (**3**), by screening the inhibitory activity of viral transcription utilizing human cell lines latently infected with HIV, namely OM10.1 from a promyelocytic cell line [30] and J-Lat from a T-lymphocytic cell line [31]. The latent HIV provirus in these cell lines were reactivated by TNF-α, which can yield a 35-fold increase in the viral gene expression [32]. The suppressive activity of globospiramine (**1**), deoxyvobtusine (**2**), and vobtusine lactone (**3**) on HIV gene transcription in stimulated HIV latently infected cell lines were also investigated (Figure 1) [33]. In addition, molecular docking studies were carried out against key canonical NF-ĸB pathway proteins, the p50/p65 heterodimer, and the inhibition of the ĸB kinase (IKK) complex [34,35] to map the putative target of the NF-ĸB inhibitory compounds investigated in this study.

## 2. Results and Discussion

Natural products have been desired as HIV therapeutic inspirations, because they are generally safe and accessible for isolation and synthesis. In the Philippines, alkaloids from medicinal plants have been continuously explored for anticancer, antioxidants, and anti-infectives; however, there are limited reports on anti-HIV derivatives. The shift to target viral protein and the cellular protein/cofactor is, by far, a promising strategy for the mode of action of a new anti-HIV drug [14,36]. Thus, our study attempted to explore new anti-HIV alkaloids that may suppress HIV production.

Cytotoxicity was first evaluated on cells of the same lineage with OM10.1 and J-Lat without the HIV infection. HL-60, the promyelocytic cell line from which OM10.1 cells were derived, and Jurkat cells, the lymphocytic cell line from which J-Lat cells were derived, were used in the preliminary screening of the alkaloids for cytotoxicity so that the range of the concentration of the compounds can be determined. Globospiramine (**1**) showed mild cytotoxicity, while deoxyvobtusine (**2**) and vobtusine lactone (**3**) were noncytotoxic (Table 1 and Figure S1).

**Figure 2:**
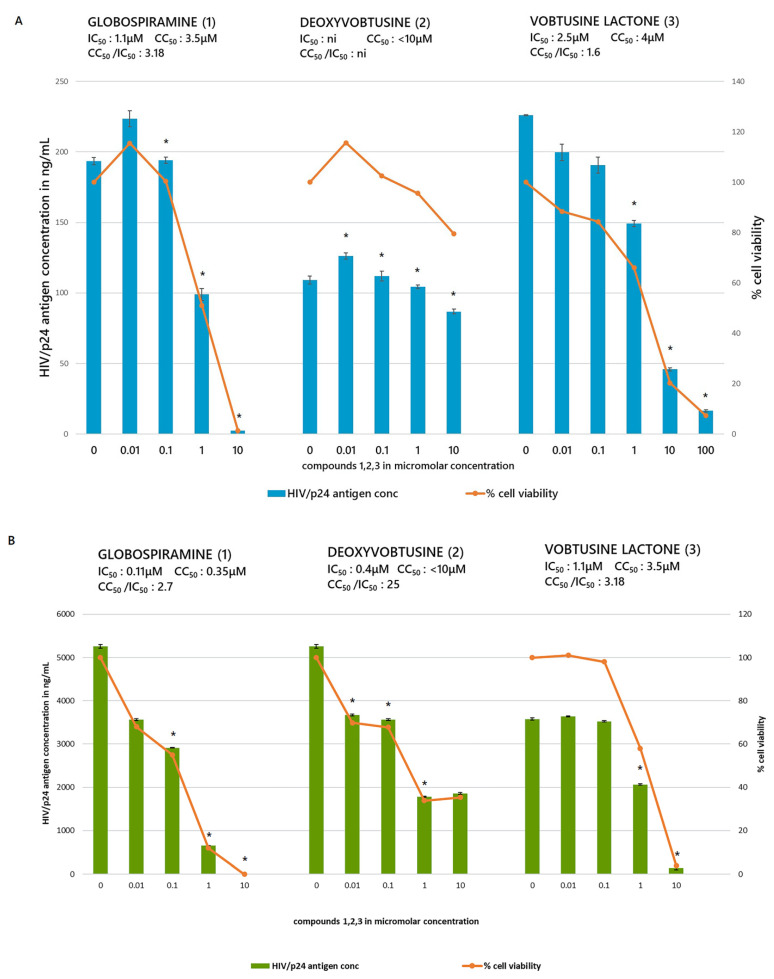
Effect of compounds in TNF-α-induced HIV latently infected cell lines (**A**) OM10.1 and (**B**) J-Lat. Cells were pretreated with varying concentrations of compounds **1**, **2**, and **3** for 4 h. Two sets were setup: one set of each cell lines was stimulated with 1-ng/mL TNF-α for HIV production and incubated for 48 h at 37 °C; the other set was not stimulated. The cells were dislodged, and the contents were spun at low speed; cell viability was determined using WST, while the supernate was tested for p24 antigen by ELISA to test for viral production. As expected, there was no significant HIV production (data not shown) in non-stimulated cells. For the other set, IC_50_ and CC_50_ were determined. Values shown are representative of the mean value ± SD of 3 independent experiments (*n* = 3). Asterisk (*) is at *p* < 0.05 for a representative experiment. Cytotoxic Concentration at 50% (CC_50_), Inhibitory Concentration at 50% (IC_50_), non-inhibitory (ni), and enzyme linked immunosorbent assay (ELISA).

**Figure 3:**
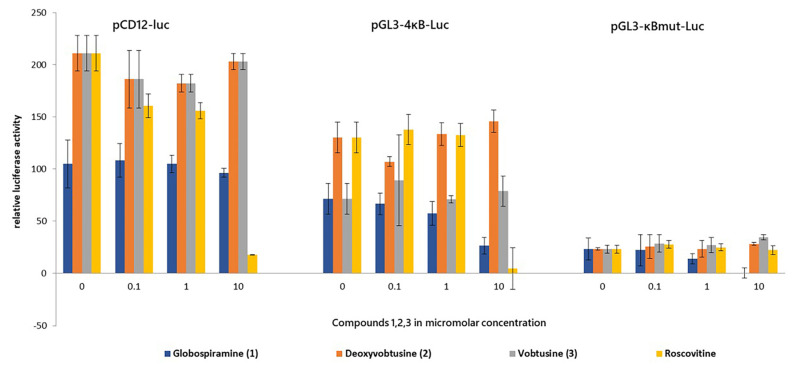
Effect of spirobisindole alkaloids **1**–**3** and roscovitine on the HIV-1 LTR-mediated transcription and on the ĸB promoter. Two hundred and ninety-three cells in a 24-well plate at 8 × 10^4^ cells per mL were transfected with the reporter plasmids: pCD12-Luc for HIV-1 LTR, pGK3-4ĸB-Luc for the wild-type ĸB promoter, and pGL3-ĸBmut-Luc for mutated ĸB sites using the Fugene 6^®^ Transfection Reagent. Cells were treated with 10-fold dilutions of the compounds **1**–**3** and roscovitine for 4 h, then treated and non-treated cells were stimulated with 5-ng/mL TNF-α for 24 h. Luminescence from treated cells of each concentration was compared against the untreated cells. Relative luciferase activity is shown as % fold activity. Values shown are representative of the mean value ± SD of 3 independent experiments (*n* = 3).

The use of HIV latently infected cell lines as models for screening compounds to suppress or inhibit HIV production has been widely used [37]. The HIV gene expression has been in low to undetectable levels but increased once stimulated. Two HIV latently infected cell lines, OM10.1 and J-Lat, were pre-treated with varying concentrations of globospiramine (**1**), deoxyvobtusine (**2**), and vobtusine lactone (**3**) and roscovitine that is known HIV inhibitor of Tat mediated transcription [38,39]. Roscovitine was found to inhibit TNF-α induced NF-ĸB activation pathway through inhibition of the IKK kinase activity and phosphorylation and degradation of IĸBα [40]. Treatment with TNF-α induced active HIV replication from OM10.1 and J-Lat cells, as previously illustrated in related studies [41,42]. During the assay experiment, reduction in HIV production was determined by measuring the p24 antigen levels with and without TNF-α stimulation. As expected, there was no HIV production in non-stimulated cells (data not shown). Based on the data, globospiramine (**1**) and vobtusine lactone (**3**) showed inhibitory activity on HIV production, with a therapeutic index (2.7 to 3.18) where a value of > is the safety numerical value (Figure 2, Table 2). This finding corroborates further on previous studies on indole alkaloids showing inhibitory activity on HIV [43] such as the Iboga type of indole plant alkaloid reduced viral production in macrophages in vitro and inhibited HIV reverse transcriptase [44], trigonoliimine A with moderate anti-HIV activity [45] and analogs of natural pimprinine alkaloid, an inhibitor of HIV integrase [46] to name a few. We observed also that deoxyvobtusine (**2**) did not demonstrate significant inhibitory activity on HIV production from TNF-α stimulated OM10.1 cells, but exhibited a remarkable therapeutic index value in TNF-α stimulated J-Lat cells. J-Lat cells are Jurkat cells containing the HIV-based lentiviral vector containing intact *gag-pol* gene and the *env* gene with a premature stop codon and an *egfp* gene replacing the *nef* gene. Unlike in stimulated OM10.1, where the HIV production is actually measured, viral reactivation in stimulated J-Lat cells is measured by GFP expression of HIV VLPs (virus-like particles) [31,37]. This suggests that deoxyvobtusine (**2**) may have an indirect effect in these VLPs, the nature of which should be further explored. The results in general suggest that compounds **1** and **3** exhibited significant inhibitory activity on HIV replication based on the p24 antigen levels. The CC_50_ values, however, imply a slight toxicity for both deoxyvobtusine (**2**) and vobtusine lactone (**3**). In order to determine whether the spirobisindole alkaloids inhibit HIV-1 gene expression, a transient luciferase assay was performed using three reporter plasmids: pCD12-luc, containing the HIV-1 long terminal repeat (LTR) promoter; pGL3-4κB-Luc, containing the four binding sites for NF-κB: pGL3-κBmut-Luc, where κB sites were mutated. Transfected 293 cells were treated with the test alkaloids separately, with or without TNF-α stimulation. Transcriptional activity was measured in this dual luciferase system. Relative luciferase activity of each reporter plasmid was determined after it was normalized for renilla luciferase reporter (Figure 3). The result of the TNF-α stimulation yielded 4–9-fold increase for upregulated gene expression in luciferase expressing reporter plasmids on HIV-LTR and NF-ĸB. Globospiramine (**1**) showed inhibitory activity on NF-ĸB but no activity on HIV-LTR gene expression, while deoxyvobtusine (**2**) showed no inhibitory activity on both HIV-LTR and NF-ĸB. Vobtusine lactone (**3**), on the other hand, showed no inhibitory activity on both HIV-LTR and NF-ĸB. We observed that only roscovitine, a known HIV Tat inhibitor, suppressed LTR-mediated transcription. Interestingly, however, globospiramine (**1**) showed inhibitory activity on NF-κB-mediated transcription. NF-κB is a key inducer of transcriptional activation in HIV gene expression [47,48]. In the cytoplasm, it exists as an inactive heterodimer p50/p65 as it is initially sequestered in the cytoplasm by the IκB family of proteins. Once stimulated, NF-κB is released, translocated to the nucleus, and binds to target sites in the enhancer region of the LTR [49]. This prompts the initial phase in the HIV transcription process. NF-κB has been extensively studied as a therapeutic target to suppress HIV replication, where inhibitory activity leads to abrogation of the release of NF-κB or to act directly to the p50 and/or p65 subunits of NF-κB to avert DNA binding at the enhancer region of the HIV-LTR [50,51,52]. Moreover, NF-ĸB does not mutate, since this is intrinsic in the T cell [53], which is the target of the virus. Weak inhibitory activity observed against pGL3-κBmut-Luc by globospiramine (**1**) suggests that other general transcription factors might be affected for its purported antiviral activity. Thus, globospiramine (**1**) is a favorable prospect as an inhibitor of NF-κB activation and can be further explored to determine the extent of inhibiting HIV in NF-κB-dependent transcriptional activity. While a number of HIV latency mechanisms related to transcriptional regulations have been indicated for epigenetic silencing and transcription repressions, our results further correlated to NF-κB suppression as a strategy to deactivate reactivated latent HIV. In support of the antagonistic activity of the alkaloid globospiramine against NF-κB, clinically established alkaloid drugs ectinascidin 743 and emetine are known to target the NF-κB pathway [54].

**Figure 4:**
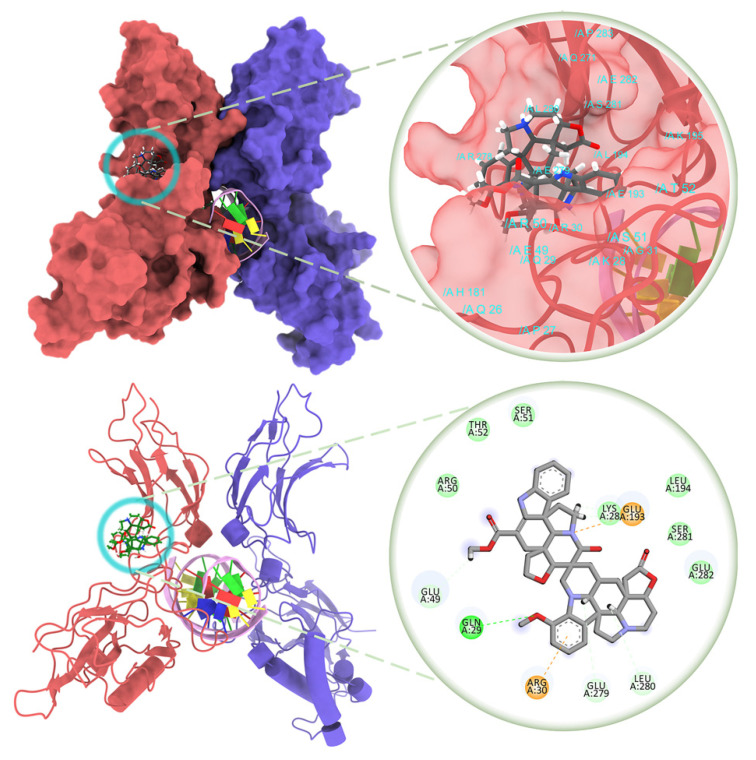
Globospiramine (**1**) docked against the p65 subunit (red) in complex with the p50 subunit (violet) of NF-κB (PDB ID: 1VKX). κB DNA was added for visualization.

**Figure 5:**
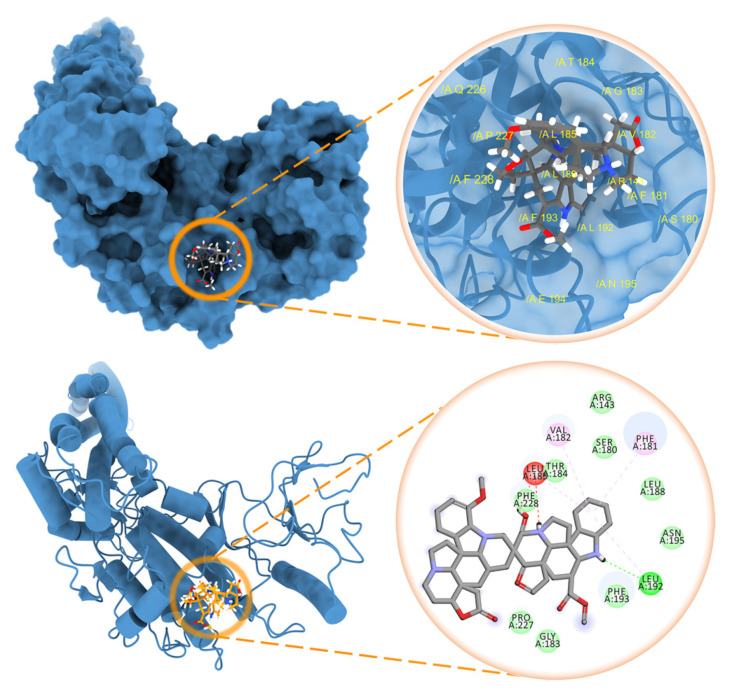
Globospiramine (**1**) docked against the activation loop of the IKKα kinase domain (PDB ID: 5EBZ).

The transcriptional inhibition mechanism of globospiramine (**1**) against NF-κB was computationally interrogated through the canonical NF-κB pathway. Blind docking against the NF-κB p50/p65 heterodimer revealed that globospiramine exhibited a stronger binding affinity of −9.2 kcal/mol than R-Roscovitine with −6.7 kcal/mol while uniquely binding to the NF-κB p65 subunit, which is the transcriptional regulatory domain of NF-κB, with several binding interactions [55]. The methoxyindoline moiety of globospiramine (**1**) exhibited a conventional hydrogen bond with Gln29 of the p65 subunit, an attractive charge interaction with Arg30, and a carbon–hydrogen bond with Glu279. An indolizine substructure interacted via van der Waals forces to Lys28 and *pi*-cation interactions with Glu193, while an indole moiety exhibited a carbon–hydrogen bond with Leu280. The anilinoacrylate moiety also formed a carbon–hydrogen bond with Glu49 (Figure 4). Most importantly, the analysis of ligand–protein interactions showed that these residues occupied by compound **1** form a p65 pocket containing residue Ser281, which is an essential phosphorylation site, since mutations of p65 Ser281 result in deficiencies in binding promoters and in the recruitment of RNA polymerase II [56]. The affinity of compound **1** to the pocket containing Ser281 of p65 was validated by the docking of spirobisindole alkaloid **1** and R-Roscovitine with a grid targeted against Ser281. Globospiramine (**1**) correspondingly conferred a higher affinity of −9.1 kcal/mol than R-Roscovitine with −6.2 kcal/mol to the targeted p65 pocket. Thus, a possible NF-κB transcriptional inhibitory mechanism of spirobisnindole alkaloid **1** is targeted against the p65 subunit. In consideration of the established mechanism of R-Roscovitine against the activity of inhibition of the κB kinase (IKK) in the canonical pathway [40], the inhibitory potential of spirobisindole alkaloid **1** and R-Roscovitine were assessed in silico against different targets in IKK. IKK is a complex composed of either or both dimers of IKKα and IKKβ, and the dimer of IKKγ, also known as the NF-κB essential modulator (NEMO) [35]. By targeting the IKK-binding site of NEMO, the NEMO-binding site of IKK subunits in the NEMO/IKKβ complex, the phosphorylation sites in the IKK activation loop, and the IKK ATP-binding site, globospiramine (**1**) and R-Roscovitine had the least binding energy requirement of −9.1 kcal/mol and −7.5 kcal/mol toward the IKKα activation loop phosphorylation sites. The indole moiety of globospiramine (**1**) bound this loop through hydrophobic *pi*-alkyl and alkyl interactions with Phe181, Val182, Leu185, and Leu192, as well as a conventional hydrogen bond with Leu192 (Figure 5). Aside from NEMO in NF-κB activation, IKK subunits are also activated through the phosphorylation of serine residues 176 and 180 in IKKα and residues 177 and 181 in IKKβ at their activation loop, rendering conformational changes in the kinase domain [35,57,58]. In the canonical NF-κB pathway, active IKK complexes phosphorylate inhibitors of κB (IκB) that are bound to NF-κB dimers, leading to the ubiquitin-mediated degradation of IκB that consequently liberates NF-κB dimers for nuclear translocation and transcription mediation [15]. The binding affinity of globospiramine (**1**) and R-Roscovitine against the IKKα subunit corroborates the in vitro antagonistic effect of TNFα-induced NF-κB activation by R-Roscovitine through the IKK autophosphorylation inhibition of both IKKα and IKKβ subunits, which was observed more on the IKKα subunit [40]. Nevertheless, the compounds exhibited binding propensities to the IKKβ phosphorylation loop congruent to the trend of in vitro antagonism of IKKβ autophosphorylation by R-Roscovitine [40]. Although the IKKβ subunit kinase activity predominantly functions in the canonical pathway, IKKα is essential for the phosphorylation of NF-κB p65 for transcriptional regulation and the accessibility modulation of chromatin [34,59]. Thus, this signifies that globospiramine (**1**) may potentially act also at the level of IKK activation and is subject to further investigation in our laboratories.

## 3. Materials and Methods

### 3.1. Test Compounds

Globospiramine (**1**), deoxyvobtusine (**2**), and vobtusine lactone (**3**) were obtained using a previously described procedure [33]. Test compounds were dissolved in dimethyl sulfoxide (DMSO) to yield a 10-mM concentration. These were serially diluted 10-fold up to 5 dilutions to yield 1-mM, 100-µM, 10-µM, and 1-µM concentrations.

### 3.2. Cell Lines and Culture Conditions

HL60 (ATCC^®^, Manassas, VA, USA, CCL-240) and Jurkat (ATCC^®^, Manassas, VA, USA, TIB-152) cells were used in preliminary screening of the alkaloids for cytotoxicity, while OM10.1 [60] and J-Lat cells [31] are HIV latently infected cells of promyelocytic origin and T-lymphocytic origin, respectively. All cell lines were maintained in RPMI 1640 (Sigma^®^, St. Louis, MO, USA) supplemented with 10% fetal bovine serum (Equitech Bio-Inc™, Kerrville, TX, USA). For the transfection assay (described below), HEK 293 cells were used. These cells were maintained in Dulbecco’s Modified Eagle’s Medium (Sigma^®^, St. Louis, MO, USA) supplemented with 10% fetal bovine serum. Cell viability was determined by the trypan blue dye test. All experiments were performed on cells during their logarithmic phase of growth.

### 3.3. Cytotoxicity Determination

In order to examine the cytotoxicity of the alkaloids to OM10.1 and J-Lat cells, solutions of the compounds at different concentrations were added to the HL-60 and Jurkat cells, which are of the same lineage as the OM10.1 and J-Lat cells, respectively, but are uninfected. Cytotoxicity assays were conducted using the WST-1 Cell Proliferation Reagent (Roche^®^, Basel, Switzerland) following the manufacturer’s directions. Briefly, the alkaloids were added to the corresponding cell lines and incubated for 24–48 h at 37 °C in an atmosphere containing 5% CO_2_. Then, two hundred microliters of the exposed cells were transferred to a microtiter plate, twenty microliters of the WST-1 reagent were added, and the cells were further incubated for up to 2 h. Absorbance was read at 450 nm, with the growth medium serving as the blank. Nonlinear regression in Microsoft Excel™ was used to determine 50% cytotoxic concentration (CC_50_).

### 3.4. Anti-HIV Assay

The indicated working concentrations of each alkaloid were added to two sets of OM10.1 and J-Lat cells. One set was stimulated with 1 ng/mL of TNF-α after 4 h of incubation with the alkaloids. All cells were incubated for 48 h at 37 °C in an atmosphere containing 5% CO_2_. The cells were collected and tested for cytotoxicity, and the CC_50_ values were determined. The supernatant was collected and tested for HIV production using the HIV p24 Antigen ELISA Kit (Rimco Corporation^®^). The 50% inhibitory concentration (IC_50_) was determined by extrapolation using nonlinear regression analysis in Microsoft Excel™. The therapeutic efficiency index was calculated as a ratio of CC_50_/IC_50_ [61].

### 3.5. Transient Transfection and Luciferase Assay

Using the Fugene-6 transfection reagent (Roche^®^, Basel, Switzerland), 293 cells were transfected with a total of 0.5 µg of plasmid DNA; pCD12-Luc for the HIV-LTR and pGL3-4κB-Luc for the four binding sites of nuclear factor-kappa B (NFκB), and pGL3-κBmut-Luc where the two tandem κB sites were mutated [61,62,63]. pUC19 was used to equalize the amount of DNA for each transfection, and pRL-TK (Promega^®^, Madison, WI, USA) was used as an internal control to monitor the efficiency of the transfection. The cells were incubated for 24–48 h at 37 °C in an atmosphere containing 5% CO_2_. After 24 h of incubation, the cells were treated with three working concentrations of each alkaloid. One set of the cells was treated with 5 ng/mL of TNF-α (Roche^®^, Basel, Switzerland) to stimulate HIV transcription. Cells were then further incubated for 48 h. Then, the cells were harvested with lysis buffer (Promega^®^, Madison, WI, USA) and subjected to a luciferase assay using the Dual Luciferase System (Promega^®^, Madison, WI, USA), both following the manufacturer’s protocols. Luciferase activity was measured using a SpectraMax ^®^ Fluorescence microplate reader (Molecular Devices, San Jose, CA, USA) [61].

### 3.6. Data Analysis

All experiments were run in triplicates, and replicability of the experimental setup was ensured. Upon treatment with the test compounds, the p24 antigen levels were compared to both unstimulated and stimulated by TNF-α cells to ensure that there is substantial decrease in the p24 antigen levels. The p24 antigen and luminescence levels in every treatment concentration were compared with stimulated TNF-α cells that were not treated with the compounds. The data were represented as the mean +/− standard error of the mean values. Student *t*-test was used, and statistical significance was set at *p* < 0.05.

### 3.7. Molecular Docking Studies

Following NF-κβ transcriptional inhibition, globospiramine (1) and the control R-Roscovitine were docked onto NF-κβ canonical pathway proteins. Compounds were first subjected to the NF-κβ p50/p65 heterodimer (PDB ID: 1VKX) [64] blind docking and the putative subunit binding was validated through p65 S281 pocket-targeted docking. Considering the established mechanism of action of R-Roscovitine against IKK activity [40], both compounds were molecularly docked onto the IKK-binding site of NEMO (PDB ID: 3BRV) [65], the NEMO/IKKβ complex (PDB ID: 3BRV) [66], both of the activation phosphorylation loops of IKKα (PDB ID: 5EBZ) [58] and IKKβ (PDB ID: 4E3C) [67], and the ATP-binding site in IKK (PDB ID: 4E3C) [68]. Molecular docking simulations were conducted in the UCSF Chimera 1.14 platform using the BFGS AutoDock Vina algorithm [69]. Protein crystal structures were retrieved as .pdb formats, in which nonstandard residues and nontarget chains were deleted. The phosphomimetic S176E and S180 in IKKα and S177E and S181E in IKKβ structures were swapped back to serine residues for phosphorylation inhibition docking. The structures of the ligands were retrieved from *V. globosa* identified by Macabeo and co-workers [33], while the structure of the control R-Roscovitine was retrieved from the synthetic analysis by Demange and coinvestigators [70]. Ligands were illustrated in ChemDraw 18.1 and built and optimized in Avogadro 1.2.0 [71]. The protein and ligands were minimized and prepared for docking through the addition of hydrogen atoms and assignment of charges according to the Gasteiger charge method using Amber’s Antechamber module [72]. The grid center and size for a binding analysis were modified based on the indicated sites of each protein in which the “flexible ligand into flexible active site” protocol through the consensus approach algorithm was employed [73,74]. Ligand–protein interactions were analyzed in BIOVIA Discovery Studio Visualizer v20.1.0.19295.

## 4. Conclusions

Among the three spirobisindole alkaloids investigated for anti-HIV activity, globospiramine (1) and vobtusine lactone (3) showed an inhibition of TNF-α-induced HIV replication in HIV latently infected cells. However, only globospiramine suppressed the NF-ĸB activation pathway based on the luciferase assay. Molecular docking studies further revealed that globospiramine antagonistically acts at the level of the NF-ĸB p65 subunit or at the level of IKK activation, which should be validated by in-depth in vitro experiments in future studies. The present study demonstrated that globospiramine potently inhibited HIV-1 latently infected OM10.1 and J-Lat cells lines compared to R-roscovitine by targeting the NF-κB pathway. Induction of the NF-ĸB cascade is a requisite process in the HIV gene transcription, and so, studies to further substantiate these findings to promote anti-HIV efficiency activity are warranted. All these observed results make spirocyclic bisindole alkaloids as a promising HIV-1 latency suppressing natural products targeting the NF-kB cascade. Our study provides new insights on a new class of alkaloid-based inhibitors of reactivated HIV latently infected cells, thereby providing an interesting strategy for anti-HIV drug discovery.

## Figures and Tables

**Table 1:** Cytotoxicity of spirobisindole alkaloids **1**–**3** in the promyelocytic and lymphocytic cell lines.

Alkaloid	HL-60 Cells (µM)	Jurkat Cells (µM)	OM10.1 Cells (µM)	J-Lat Cells (µM)
Globospiramine (**1**)	0.75	0.50	0.70	0.350
Deoxyvobtusine (**2**)	>56.940	>56.940	>10.0	>10.0
Vobtusine lactone (**3**)	8.0	11.0	0.250	0.100

The data shown are the mean values ± SD of triplicate experiments (*n* = 3). Cytotoxic Concentration at 50% (CC_50_). Values shown are representative of the mean value of triplicate data.

**Table 2:** CC_50_, IC_50_ and index CC_50_/IC_50_ of spirobisindole alkaloids **1**–**3** in the TNF-α induced OM10.1 and J-Lat cell lines.

	OM10.1 Cells			J-Lat Cells		
Alkaloid	CC_50_ (µM)	IC_50_ (µM)	CC_50_/IC_50_	CC_50_ (µM)	IC_50_(µM)	CC_50_/IC_50_
Globospiramine (**1**)	3.5	1.1	3.18	0.35	0.11	2.7
Deoxyvobtusine (**2**)	>10	ni	ni	>10	0.4	25
Vobtusine lactone (**3**)	4	2.5	1.6	3.5	1.1	3.18
Roscovitine	8	2.75	2.90	0.45	0.25	1.8

Values shown in the table are representative of the mean values ± SD of triplicate experiments (*n* = 3). Cytotoxic Concentration at 50% (CC_50_), and Inhibitory Concentration at 50% (IC_50_).

## Data Availability

Data is contained within the article.

## Supplementary Materials

The following supporting information can be downloaded online, Figure S1: Cytotoxicity of spirobisindole alkaloids **1**–**3** in promyelotic and lymphocytic cell lines.

## Sample Availability

Not available.
