# Correction: de Jesus et al. *Voacanga globosa* Spirobisindole Alkaloids Exert Antiviral Activity in HIV Latently Infected Cell Lines by Targeting the NF-κB Cascade: In Vitro and In Silico Investigations. *Molecules* 2022, *27*, 1078

**DOI:** 10.3390/molecules29030626

**Published:** 2024-01-29

**Authors:** Ma. Sheila M. de Jesus, Allan Patrick G. Macabeo, John Donnie A. Ramos, Von Novi O. de Leon, Kaori Asamitsu, Takashi Okamoto

**Affiliations:** 1 The Graduate School, University of Santo Tomas, España Blvd., Manila 1015, Philippines; jaramos@ust.edu.ph; 2 Department of Biological Sciences, College of Science, University of Santo Tomas, España Blvd., Manila 1015, Philippines; vonnovi.deleon.sci@ust.edu.ph; 3 Laboratory for Organic Reactivity, Discovery and Synthesis (LORDS), Research Center for Natural and Applied Sciences, University of Santo Tomas, España Blvd., Manila 1015, Philippines; agmacabeo@ust.edu.ph; 4 Molecular Diagnostics and Therapeutics Laboratory, Research Center for Natural and Applied Sciences, University of Santo Tomas, España Blvd., Manila 1015, Philippines; 5 Department of Molecular and Cellular Biology, Graduate School of Medical Sciences, Nagoya City University, Nagoya 4678601, Japan; asamitsu@med.nagoya-cu.ac.jp (K.A.); takoka221@gmail.com (T.O.)

## Error in Article Title

There was an error in the original publication [1]. “NF-kB” should be “NF-κB”.

## Text Correction

There was an error in the original publication [1]. **The method stated in “3.4 Anti-HIV Assay” is a duplicate entry of “3.3 Cytotoxicity Determination”**.

A correction has been made to **3.4**, *Anti-HIV Assay*:

*CORRECTED paragraph:**

The indicated working concentrations of each alkaloid were added to two sets of OM10.1 and J-Lat cells. One set was stimulated with 1 ng/mL of TNF-α after 4 h of incubation with the alkaloids. All cells were incubated for 48 h at 37 °C in an atmosphere containing 5% CO_2_. The cells were collected and tested for cytotoxicity, and the CC_50_ values were determined. The supernatant was collected and tested for HIV production using the HIV p24 Antigen ELISA Kit (Rimco Corporation^®^). The 50% inhibitory concentration (IC_50_) was determined by extrapolation using nonlinear regression analysis in Microsoft Excel™. The therapeutic efficiency index was calculated as a ratio of CC_50_/IC_50_ [63].

As a result of the above changes, the citation order in the article has been modified, ref. 63 changed into ref. 61.

The authors apologize for any inconvenience caused and state that the scientific conclusions are unaffected. This correction was approved by the Academic Editor. The original publication has also been updated.
