# Supplementary material for: Voacanga globosa Spirobisindole Alkaloids Exert Antiviral Activity in HIV Latently Infected Cell Lines by Targeting the NF-κB Cascade: In Vitro and In Silico Investigations
Source: Molecules. 2022 Feb 5;27(3):1078. doi: 10.3390/molecules27031078 (PMC8840767; doi:10.3390/molecules27031078)
Supplement: Supplementary file 1 [file molecules-27-01078-s001.zip › molecules-1483135-Supplementary.pdf]

SUPPLEMENTARY INFORMATION

Voacanga globosa Spirobisindole Alkaloids Exert Antiviral Activity in HIV Latently Infected Cell Lines by Targeting the NF-kB Cascade: In Vitro and In Silico Investigations

Ma. Sheila M. de Jesus[1,2,*], Allan Patrick G. Macabeo[3], John Donnie A. Ramos[1,2,4], Von Novi O. de Leon[2,3], Kaori Asamitsu[5] and Takashi Okamoto[5]

[1]The Graduate School, University of Santo Tomas, España Blvd., Manila, 1015 Philippines; jaramos@ust.edu.ph

[2]Department of Biological Sciences, College of Science, University of Santo Tomas, España Blvd., Manila, 1015 Philippines; vonnovi.deleon.sci@ust.edu.ph

[3]Laboratory for Organic Reactivity, Discovery and Synthesis (LORDS), Research Center for Natural and Applied Sciences, University of Santo Tomas, España Blvd., Manila 1015 Philippines; agmacabeo@ust.edu.ph

[4]Molecular Diagnostics and Therapeutics Laboratory, Research Center for Natural and Applied Sciences, University of Santo Tomas, España Blvd., Manila 1015 Philippines

[5]Department of Molecular and Cellular Biology, Graduate School of Medical Sciences, Nagoya City University, Nagoya 4678601, Japan; asamitsu@med.nagoya-cu.ac.jp (K.A.); takoka221@gmail.com (T.O.)

* Correspondence: mmdejesus@ust.edu.ph; Tel.: +63-2-87315728

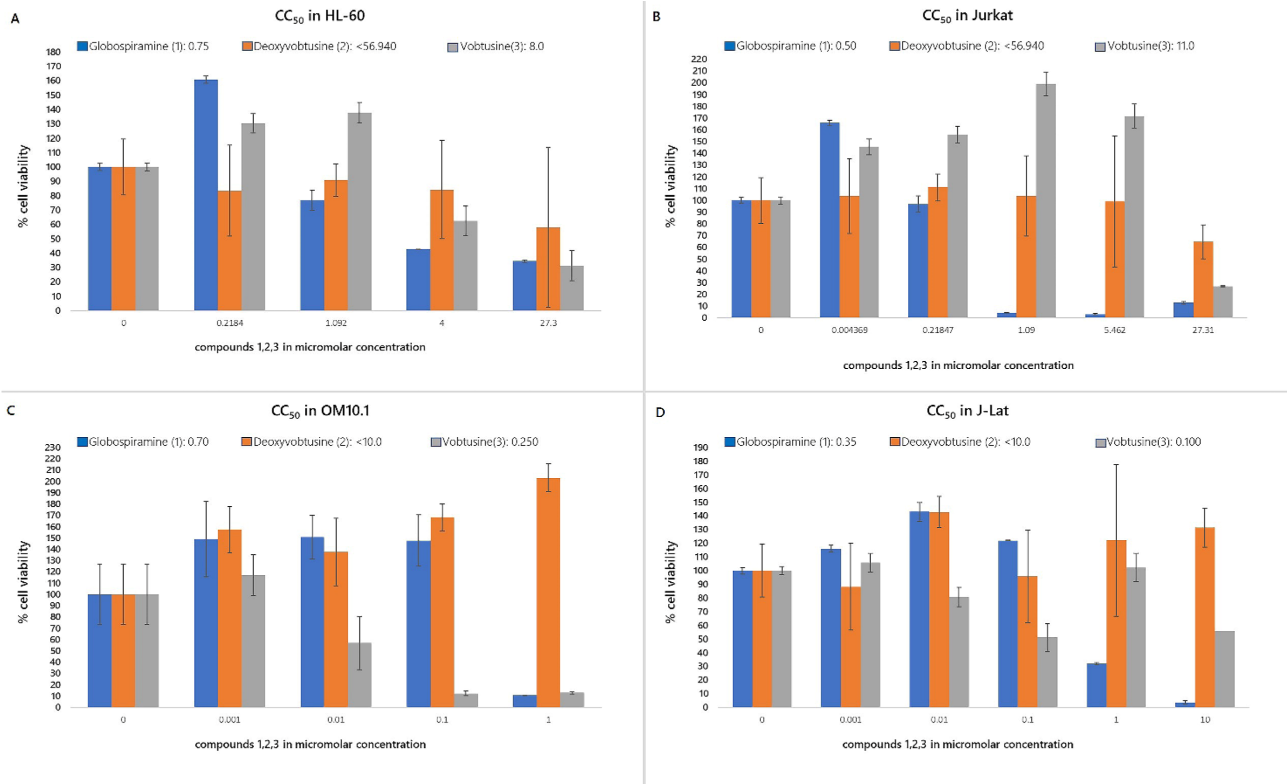

Figure S1. Cytotoxicity of spirobisindole alkaloids **1**–**3** in promyelotic and lymphocytic cell lines.
